# P-1557. Using cefazolin and ceftriaxone via automated susceptibility predicts susceptibility to oral cefpodoxime but not cefuroxime in *Enterobacterales*

**DOI:** 10.1093/ofid/ofae631.1724

**Published:** 2025-01-29

**Authors:** Joseph Sassine, Cindy B McCloskey, Bryan White, Nayle Ibragimova, Denise Robison, Steven Pan, Maria Alkozah, Emily A Siegrist

**Affiliations:** University of Oklahoma Health Sciences Center, Oklahoma City, Oklahoma; University of Oklahoma College of Medicine, Oklahoma City, OK; University of Oklahoma Medical Center, Oklahoma City, Oklahoma; OU Health, Oklahoma City, Oklahoma; OU Health, Oklahoma City, Oklahoma; Hudson College of Public Health, Oklahoma City, Oklahoma; OU Health, Oklahoma City, Oklahoma; OU Health, Oklahoma City, Oklahoma

## Abstract

**Background:**

Current CLSI recommendations suggest using cefazolin to predict susceptibilities of oral cephalosporins for uncomplicated UTI (uUTI) due to *Escherichia coli*, *Klebsiella pneumoniae*, or *Proteus mirabilis*. Most laboratories do not conduct routine susceptibility testing of oral cephalosporins as they are not available on many automated panels. However, there are limited data which evaluate ceftriaxone and cefazolin categorical agreement with oral cefpodoxime and cefuroxime. This study assessed agreement between ceftriaxone or cefazolin via MicroScan with cefuroxime or cefpodoxime via disk diffusion.

**Figure:**
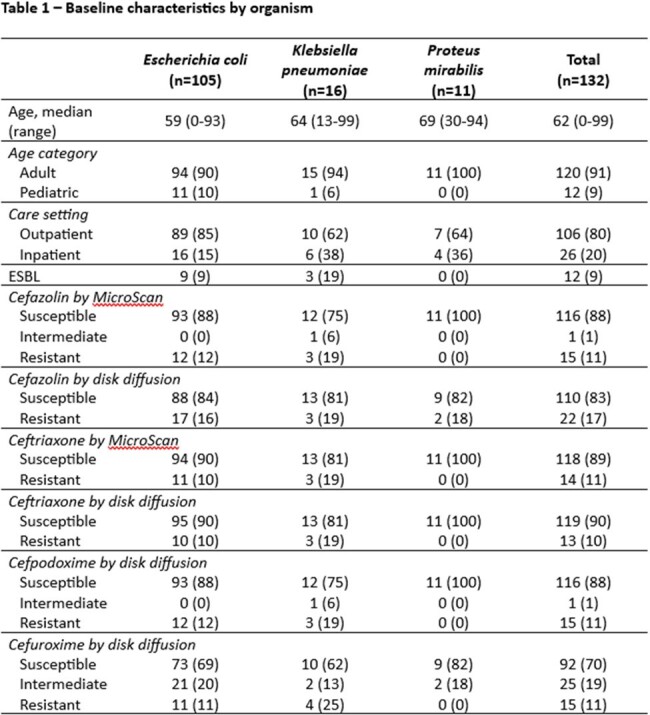

**Methods:**

We collected *E. coli*, *K. pneumoniae*, or *P. mirabilis* isolates from urine cultures in a single center between 2/2024 and 3/2024. Identification was performed using MALDI-TOF and susceptibility testing was conducted overnight broth dilution panels (MicroScan, Beckman Coulter) and disk diffusion for cefazolin and ceftriaxone and by disk diffusion for cefuroxime and cefpodoxime. The primary outcome was categorical agreement assessed by Fleiss’ kappa of automated panel susceptibility for cefazolin and ceftriaxone with cefpodoxime and cefuroxime. Secondary outcomes included disk diffusion categorical agreement and rates of very major, major, and minor errors.

**Figure:**
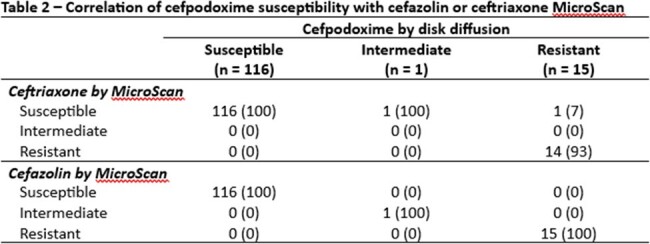

**Results:**

We included 132 isolates, 105 (79.5%) *E. coli*, 16 (12.1%) *K. pneumoniae*, and 11 (8.3%) *P. mirabilis*. Ceftriaxone had slightly lower categorical agreement with cefpodoxime than cefazolin (98% vs 100%). Furthermore, very major errors were more common when using ceftriaxone to predict susceptibility (7% vs 0%). Ceftriaxone and cefazolin both were poor predictors of cefuroxime susceptibility (80% vs 80%).

**Figure:**
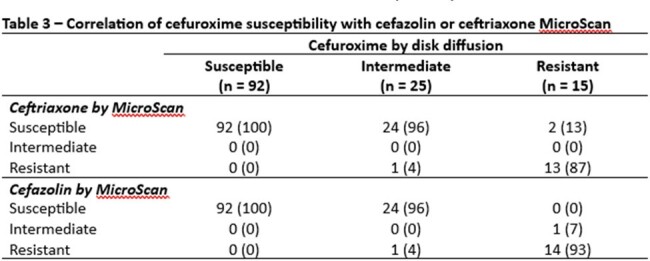

**Conclusion:**

In this sample, both cefazolin and ceftriaxone had high categorical agreement with cefpodoxime; however, cefazolin resulted in fewer very major errors. On the other hand, cefazolin and ceftriaxone had much lower categorical agreement with cefuroxime, mostly driven by isolates intermediate to cefuroxime but susceptible to cefazolin and ceftriaxone. Our data support the CLSI recommendations to use cefazolin over ceftriaxone to predict susceptibility of cefpodoxime but do not support the use of cefazolin to predict activity of oral cefuroxime for uUTI.

**Figure:**
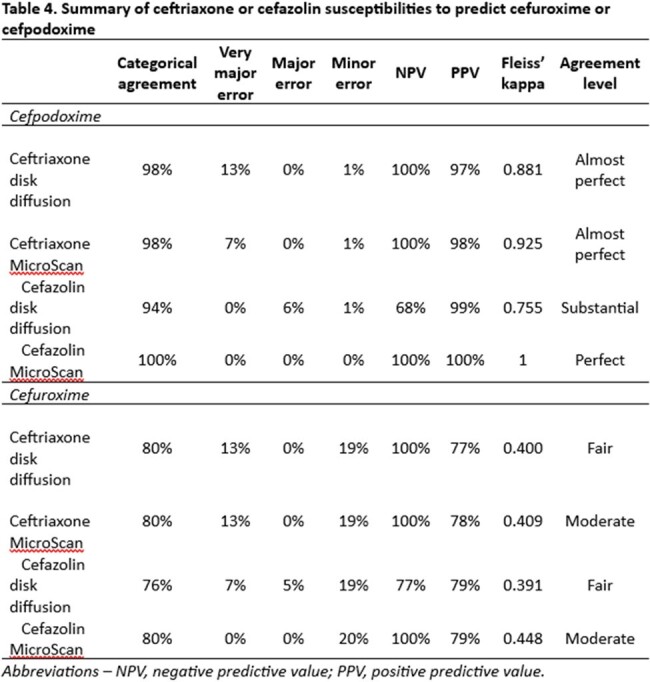

**Disclosures:**

**Joseph Sassine, MD**, Ansun BioPharma: Grant/Research Support|Cidara Therapeutics: Grant/Research Support|Community Infusion Solutions: Grant/Research Support|F2G: Grant/Research Support|Shionogi: Grant/Research Support **Bryan White, PharmD, BCPS, BCIDP**, Melinta: Advisor/Consultant|Shionogi: Grant/Research Support

